# Quantitative Structure–Activity Relationship Approaches for Exploring the Anticancer Potential of Flavonoids

**DOI:** 10.1002/cbdv.71728

**Published:** 2026-09-27

**Authors:** Mukta Gupta, Shanu Priya, Javed Ahmad, Kasim Sakran Abass, Awanish Mishra

**Affiliations:** ^1^ School of Pharmaceutical Sciences Lovely Professional University Punjab India; ^2^ Department of Pharmaceutics College of Pharmacy Najran University Najran Saudi Arabia; ^3^ Department of Physiology Biochemistry, and Pharmacology College of Veterinary Medicine University of Kirkuk Kirkuk Iraq; ^4^ Department of Pharmacology and Toxicology National Institute of Pharmaceutical Education and Research (NIPER) – Guwahati Kamrup Assam India

**Keywords:** anticancer activity, apoptosis and autophagy, chemoinformatics, drug discovery and development, flavonoids, molecular docking, PI3K/Akt signaling, polyphenols, quantitative structure–activity relationship (QSAR), structure–activity relationship (SAR)

## Abstract

Flavonoids are structurally diverse polyphenolic compounds widely distributed in fruits, vegetables, grains, and beverages, with considerable potential as anticancer agents. Their pleiotropic activities involve modulation of key cancer hallmarks, including oxidative stress, cell‐cycle progression, apoptosis, autophagy, angiogenesis, invasion, and metastasis. These effects are mediated through multiple signaling pathways, including PI3K/Akt, MAPK, NF‐κB, and STAT3. Understanding the relationship between flavonoid structure and biological activity is therefore essential for rational optimization of flavonoid‐based therapeutics. Structure–activity relationship (SAR) and quantitative structure–activity relationship (QSAR) approaches provide systematic frameworks for correlating structural and physicochemical features, including hydroxylation, glycosylation, prenylation, electronic distribution, steric properties, and lipophilicity, with anticancer activity and target interactions. This review summarizes advances in flavonoid identification and characterization and critically examines computational approaches used to investigate their anticancer potential, including molecular docking, linear and nonlinear QSAR modeling, molecular similarity analysis, topological descriptors, semiempirical calculations, density functional theory, molecular dynamics simulations, and Free–Wilson analysis. Particular emphasis is placed on integrating computational predictions with experimental validation and addressing challenges related to pharmacokinetics, bioavailability, selectivity, and translation. Collectively, SAR/QSAR‐guided strategies offer valuable tools for elucidating structure–activity relationships and accelerating the rational discovery and optimization of flavonoid‐based anticancer candidates.

## Graphical abstract

QSAR‐based analysis of flavonoids to identify structural determinants of anticancer activity and prioritize promising candidates.

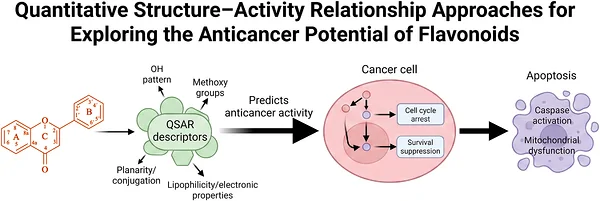

## 1 Introduction

Cancer is a complex multifactorial disease, involving a dynamic interplay between genetic, epigenetic, and environmental factors; hence, it remains a major global health burden [1, 2, 3, 4, 5]. The overall incidence of cancer has increased substantially over the past two decades, although therapeutic advances have improved survival rates for certain malignancies. The rising global incidence emphasizes the continuous demand for novel, effective chemo‐preventive and targeted therapeutic agents [6].

In this context, naturally occurring phytochemicals, particularly flavonoids, have attracted considerable attention as potential anticancer agents because of their broad spectrum of biological activities and structural diversity. Mechanistically, flavonoids exhibit a unique dual effect on oxidative homeostasis; while acting as powerful antioxidants in normal physiological tissues, they induce selective pro‐oxidant activity in malignant cells, elevating intracellular reactive oxygen species (ROS) levels to trigger caspase‐dependent intrinsic apoptosis and cell cycle arrest at the G_2_/M or G_1_/S phases [7, 8]. Flavonoids exert anticancer effects through multiple mechanisms, including the modulation of oxidative stress, regulation of apoptotic, and autophagic pathways, inhibition of angiogenesis, and interference with oncogenic signaling cascades. Despite substantial experimental evidence supporting their anticancer potential, the translation of flavonoids into clinically viable agents remains challenging. These limitations arise from their extensive structural diversity, variability in substitution patterns, multitarget behavior, and complex pharmacokinetic profiles [9, 10].

Quantitative structure–activity relationship (QSAR)‐based methodologies offer powerful computational tools for systematically correlating the chemical features of flavonoids with their biological activities. QSAR approaches enable the identification of critical structural determinants, such as hydroxylation patterns, conjugation, lipophilicity, steric factors, and electronic properties, that govern anticancer efficacy, target affinity, and pharmacokinetic behavior. Integration of QSAR modeling with molecular docking, molecular descriptors, and chemoinformatics tools facilitates rational lead optimization, virtual screening, and prediction of biological activity, thereby reducing experimental cost and time while improving drug discovery efficiency [11, 12]. Consequently, QSAR‐driven analyses provide valuable insights into structure‐based anticancer drug design and support the development of flavonoid‐derived scaffolds as promising candidates for future anticancer therapies [13].

Despite the growing body of experimental and computational data, several critical gaps hinder the rational clinical translation of flavonoid‐based anticancer agents. The existing literature is highly fragmented, with most studies focusing on isolated biological activities, single cancer models, or individual molecular targets, thereby failing to capture the inherent polypharmacological nature of flavonoids. Furthermore, structure–activity relationships have often been described qualitatively, with limited integration of QSAR modeling, molecular docking, and mechanistic validation to elucidate how specific structural attributes govern anticancer activity and pathway selectivity. Another major limitation is the inadequate exploration of resistance mechanisms, particularly apoptosis resistance, autophagy‐mediated survival, and compensatory oncogenic signaling, which are central challenges in contemporary cancer therapies. Consequently, a result, a critical gap persists in the development of integrative frameworks that link flavonoid chemical diversity with computational modeling and multi‐pathway anticancer mechanisms. Addressing these limitations is essential to transition flavonoid research from descriptive bioactivity studies to predictive, mechanism‐oriented, and rational anticancer drug discovery strategies.

## 2 Phytoconstituents and Cancer: A Close Relationship

Natural products derived from living organisms have long played a pivotal role in human health and disease management [14]. Medicinal plants have been extensively utilized worldwide for the treatment of a broad spectrum of disorders, including inflammation, ulcers, diabetes, cardiovascular diseases, neurological disorders, cancer, respiratory ailments, and rheumatoid arthritis. These plant‐derived products encompass a diverse range of phytochemicals, including alkaloids, terpenes, flavonoids, glucosinolates, phenolic compounds, saponins, tannins, and resins. It is important to note that, although these natural products are not drugs per se, they constitute a rich reservoir of bioactive molecules that serve as valuable starting points for modern drug discovery and development.

Despite the considerable therapeutic potential of phytoconstituents, their direct translation into clinically approved drugs remains limited, contributing to a decline in pharmaceutical interest in natural product–based drug development. Nevertheless, according to the World Health Organization (WHO), approximately 80% of the global population continues to rely on herbal medicines for primary healthcare, underscoring their enduring medical and socioeconomic relevance. Although extensive research has demonstrated the anticancer potential of numerous plant‐derived compounds, their clinical application remains rare. Many of these bioactive agents are secondary metabolites that can act as lead compounds and can be structurally optimized through chemical modification to enhance their bioavailability, stability, and therapeutic efficacy against various cancer types [15, 16, 17].

Flavonoids are one of the most abundant and structurally diverse classes of phytochemicals among plant secondary metabolites. To date, more than 8 000 flavonoids have been identified in natural sources. Based on their chemical structures, flavonoids are classified into several subclasses, including flavans, chalcones, flavanonols, flavanones, flavones (anthoxanthins), neoflavonoids, isoflavonoids, and anthocyanidins. These compounds exhibit a wide range of biological activities, including antioxidant, anti‐inflammatory, and anticancer effects [7, 18, 19, 20, 21, 22]. This review focuses on flavonoid chemistry and highlights quantitative structure–activity relationship (QSAR)‐based approaches for elucidating their anticancer potential and facilitating the rational development of flavonoid‐based anticancer agents.

## 3 Insight Into Various Flavonoids

**FIGURE 1:**
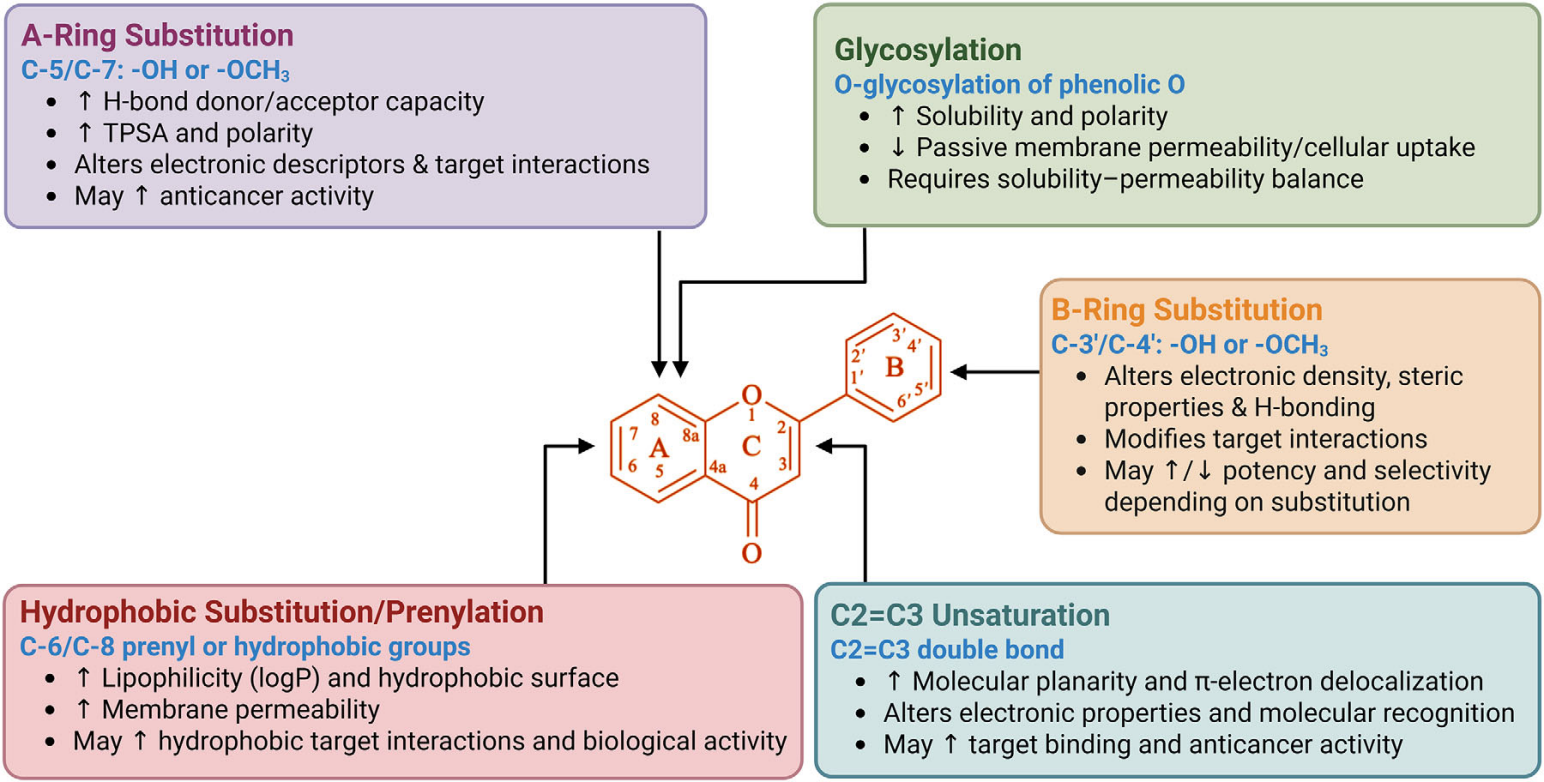
General flavonoid scaffold highlighting key structural modification sites, including hydroxylation, glycosylation, prenylation, and ring substitutions, and their influence on physicochemical properties, molecular descriptors, and QSAR‐based prediction of anticancer activity [27, 28, 29, 30, 31]. Created in BioRender. Mishra, A. (2026) https://BioRender.com/js4utum.

Flavonoids are plant‐derived secondary metabolites characterized by a basic flavan skeleton consisting of a 15‐carbon phenylpropanoid framework with a common C6–C3–C6 structure (Figure 1) [23]. These compounds play crucial roles in determining the color, flavor, and overall physiology of plants. In the human diet, flavonoids are primarily consumed through fruits and vegetables; however, they are also abundantly present in seeds, legumes, nuts, grains, spices, tea, wine, and various other plant‐based foods, although at varying concentrations. Epidemiological and experimental studies suggest that diets rich in flavonoids exert nutraceutical benefits by protecting against diseases associated with reduced lifespan, thereby contributing to an improved life expectancy [24]. Beyond their role in plant survival, flavonoids exhibit a wide range of biological activities, including antioxidant, anti‐inflammatory, and anticancer effects, which are highly relevant to human health [25, 26].

## 4 Structure and Biological Sources of Flavonoids

Flavonoids are widely distributed polyphenolic compounds found in diverse plant species, with their concentration and chemical forms varying depending on the plant type, tissue, and developmental stage. Structurally, flavonoids may occur in plants as aglycones or in conjugated forms, such as methylated or glycosylated derivatives, which significantly influence their solubility, stability, bioavailability, and biological activity [27, 28]. The chemical structures and botanical sources of the selected flavonoids exhibiting notable anticancer activity are summarized in Table 1.

**TABLE 1:** Chemical structure and biological source of flavonoids.

Flavonoid	Structure	Biological source	Family	Reference
Fisetin	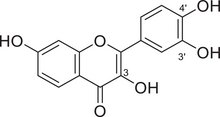	*Rhus cotinus* L. *Acacia berlandieri* Benth. *Acacia greggii A. Gray* *Elaeagnus indica* Serv. Bull.	Anacardiaceae Fabaceae Fabaceae Elaeagnaceae	[32, 33, 34]
Quercetin	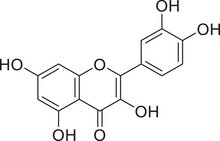	*Morus alba* L. *Camellia sinensis* (L.) Kuntze *Allium fistulosum* L. *Apium graveolens* L. *Centella asiatica* (L.) Urb. *Moringa oleifera* *Asparagus officinalis* L. *Allium cepa L*.	Moraceae Theaceae Liliaceae Apiaceae Apiaceae Moringaceae Liliaceae Amaryllidaceae	[35, 36, 37, 38]
Kaempferol	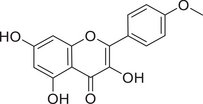	*Ginkgo biloba* L. *Rosmarinus officinalis* L *Crocus sativus* L. *Aloe vera* (L.) Burm.f. *Phyllanthus emblica* L. *Foeniculum vu*l*gare* Mill. *Solanum nigrum* L.	Ginkgoaceae Lamiaceae Iridaceae Asphodelaceae Euphorbiaceae Apiaceae Solanaceae	[39, 40, 41, 42]
Hesperidin	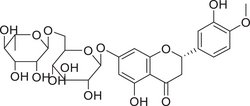	*Citrus sinensis* (L.) *Zanthoxylum avicennae* (Lam.) DC. Commiphora myrrha (Nees) Engl. *Citrus reticulate* Blanco	Rutaceae Rutaceae Burseraceae Rutaceae	[43, 44, 45]
Apigenin	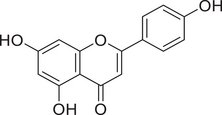	*Matricaria recutita* L. *Ginkgo biloba* L. *Achillea abrotanoides* (Vis.) *Tanacetum achillea* Sch.Bip. *Artemisia abrotanum* L. *Hypericum perforatum* L	Asteraceae Ginkgoaceae Asteraceae Asteraceae Asteraceae Clusiaceae	[46, 47, 48]
Luteolin	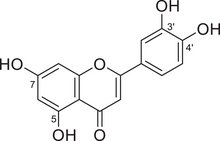	*Capsicum annuum* L. *Apium graveolens* L. *Daucus carota* L. *Theobroma cacao* L. *Thymus vulgaris* L. *Olea europaea* L. *Punica granatum* L.	Solanaceae Apiaceae Apiaceae Malvaceae Lamiaceae Oleaceae Punicaceae	[49, 50, 51, 52, 53, 54]
Icaritin	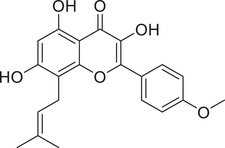	*Epimedii herba* *Epimedium brevicornum*	Berberidaceae	[55]
Myricetin	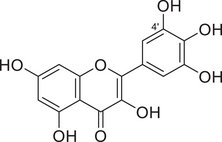	*Comptonia peregrina* (L.) Coult. *Morella cerifera* (L.) *Rosa damascene* *Pistacia lentiscus* L. *Urtica dioica* L. (nettle) *Polygonum bellardii* All. *Euphorbia tirucalli L*. *Cyperus rotundus* L.	Myricaceae Myricaceae Rosaceae Anacardiaceae Urticaceae Polygonaceae Euphorbiaceae Cyperaceae	[56, 57, 58]

## 5 Quantitative Structure–Activity Relationship of Anticancer Flavonoids

Structure–activity relationship (SAR) and quantitative structure–activity relationship (QSAR) have emerged as powerful computational approaches to predict the anticancer activity of flavonoids by correlating their chemical structures with biological responses. The prediction of pharmacological efficacy is inherently complex, as variations in substitution, such as the position and number of substituents, the presence of isoprenoid units, and the nature of functional groups, significantly influence cytotoxicity and, consequently, anticancer potential [59, 60]. Consistent observations have been reported by Aidiel et al., who highlighted the critical role of molecular configuration and substitution patterns in modulating protein–ligand interactions and cytotoxic responses [61].

Various QSAR methodologies, including multiple linear regression, partial least squares analysis, topological modeling, artificial neural networks, and Free–Wilson analysis, have been employed to identify the structural determinants underlying flavonoid‐mediated pharmacological activity [62]. However, many existing models still lack sufficient mechanistic interpretability and predictive robustness. The integration of cheminformatics tools enables the transformation of complex chemical datasets into meaningful descriptors, which, when combined with molecular docking studies, facilitates the prediction of the target affinity and biological efficacy of candidate molecules [63].

Several studies have exemplified the application of QSAR modeling in flavonoid‐based anticancer research. Qian et al. developed and validated a QSAR model for flavonoid–metal complexes using quantum chemical descriptors as independent variables and IC_50_ values as dependent variables, providing a theoretical framework for predicting their anticancer activity against HepG2 cells [64]. In another study, a 2D‐QSAR model was constructed to elucidate the relationship between flavonoid‐mediated inhibition of P‐glycoprotein in KB/MDR1 cells and solvation energy–based descriptors, revealing that specific substitution patterns are critical for effective P‐glycoprotein inhibition [65]. Furthermore, combined 3D‐QSAR and molecular docking analyses of flavonoid analogs identified their potential as tankyrase inhibitors, although further optimization is required to develop novel flavonoid‐derived anticancer scaffolds [11].

QSAR modeling also aids in the rational selection and optimization of physicochemical properties of compounds that are crucial for cytotoxic activity [66]. For instance, the computational evaluation of flavonoids as nitric oxide synthase (NOS) inhibitors demonstrated that more negative ionization energy and higher electrophilicity indices are associated with enhanced inhibitory efficacy [67]. These findings underscore the importance of comprehensive QSAR‐based investigations for the predictive identification of potent anticancer agents.

Comparative QSAR analyses further revealed that flavonoids do not exhibit uniform anticancer activity, and even minor structural modifications can result in pronounced differences in biological response [68]. Flavones and flavonols bearing hydroxyl groups at the C‐6, C‐7, or C‐4 positions often display enhanced antiproliferative activity due to their ability to form hydrogen bonds, π–π stacking interactions, and favorable electronic complementarity with target proteins. However, excessive hydroxylation may negatively affect membrane permeability and metabolic stability, thereby reducing overall efficacy [69]. Prenylated flavonoids generally exhibit higher anticancer activity than their non‐prenylated counterparts, an effect attributed to increased lipophilicity and improved target accessibility, as supported by QSAR‐derived hydrophobic descriptors [68]. In contrast, glycosylated flavonoids often exhibit reduced *in vitro* activity, reflecting a trade‐off between enhanced solubility and limited cellular uptake.

Collectively, these insights highlight the necessity of QSAR‐based prioritization strategies to distinguish promising flavonoid scaffolds from structurally similar yet biologically fewer effective analogs. Such integrative computational approaches are essential for advancing flavonoid‐based anticancer drug discovery toward rational, predictive, and mechanism‐oriented design frameworks [70].

## 6 Identification of Flavonoids

Accurate identification and quantification of flavonoids and total phenolic content in plant materials are critical for phytochemical characterization and subsequent biological evaluations. To this end, several qualitative and quantitative methods, including the Folin–Ciocalteu assay and aluminium chloride colorimetric method, are commonly employed to estimate the total phenolic and flavonoid content in different plant parts [71]. In addition, a range of classical chemical tests has been used for the preliminary detection of flavonoids in plant extracts. To systematically structure the analytical and structural profiling of these phytoconstituents, this section is categorized into the following subsections.

### 6.1 Structural Diversity: Aglycones, *O*‐Glycosides, *C*‐Glycosides, and Derivatives

Flavonoids are a chemically heterogeneous group of plant‐derived polyphenols that occur in diverse structural forms, including free aglycones, *O*‐glycosides, *C‐*glycosides, and various methylated, hydroxylated, and prenylated derivatives [72]. The differentiation of these structurally diverse glycosides relies on their unique physicochemical behaviors and specific spectroscopic or chromatographic fragmentation patterns [73]. Free aglycones are non‐polar to moderately polar compounds that exhibit distinct UV–visible absorption maxima corresponding to the core flavonoid skeleton and undergo straightforward chromatographic retention in reverse‐phase systems. In contrast, glycosylated flavonoids exhibit increased water solubility and altered retention behavior. Structurally, *O*‐glycosides feature a hemiacetal C–O linkage between the sugar and flavonoid hydroxyl group, which readily undergoes acid‐ or enzyme‐catalyzed hydrolysis, yielding characteristic neutral losses of sugar units in mass spectrometry. Conversely, *C*‐glycosides possess a direct C─C covalent bond between the anomeric sugar carbon and the flavonoid aglycone core, offering high thermodynamic stability and resisting standard enzymatic and acid hydrolysis; hence, they do not exhibit clean glycosidic cleavage [74, 75].

### 6.2 Classification by Flavonoid Subclasses

Flavonoids are classified into several subclasses, such as flavones, flavonols, flavanones, isoflavones, anthocyanidins, and chalcones, based on variations in the oxidation state of the heterocyclic C‐ring, degree of unsaturation, and position of phenyl substitution [73]. These structural variations markedly influence their physicochemical properties, including polarity, solubility, stability, ionization behavior, and chromophoric characteristics, which, in turn, determine their analytical detectability and separation efficiency [76]. For instance, comparative structural profiling of flavonoids subclasses is governed by spectroscopic detection, such as isoflavone, which can be differentiated by H‐2 and C‐2 shifts, which differ from the H‐3 and C‐3 shifts of flavone in nuclear magnetic resonance (NMR). Flavonone, without C2 = C3 unsaturation, exhibits a typical chemical shift [77].

### 6.3 Analytical Strategies and Subclass Differentiation

Structural modification, such as glycosylation and methylation, can alter the UV–visible absorption maxima, reduce chromatographic retention, and complicate spectral interpretation. Furthermore, isomerism among flavonoid subclasses often results in overlapping signals when conventional analytical methods are applied. Consequently, the reliable identification and characterization of flavonoids require a systematic, stepwise analytical strategy that integrates preliminary qualitative screening with advanced chromatographic and spectroscopic techniques. This approach enables accurate subclass differentiation, discrimination between isomeric forms, and precise determination of substitution patterns, providing a robust chemical foundation for subsequent biological evaluation and QSAR analysis [78]. In this context, Vukics and Guttman (2010) indicated that the differentiation of distinct flavonoid glycoside subclasses is based on fragmentation peaks in their negative ion spectra using hyphenated techniques, such as liquid chromatography–mass spectrometry (LC‐MS), which provides adequate information about positional isomers [79].

### 6.4 Advanced Chromatographic and Spectroscopic Profiling of Flavonoids

In addition to classical assays, modern chromatographic and spectrophotometric techniques have been extensively employed for the identification and quantification of flavonoids in plant sources. Given the occurrence of flavonoids in multiple isomeric forms, their comprehensive characterization cannot be achieved using a single analytical technique, underscoring the need for high‐resolution multidimensional analytical methods [80]. In this context, hyphenated techniques such as LC–MS, liquid chromatography‐tandem mass spectrometry (LC–MS/MS), and liquid chromatography–nuclear magnetic resonance (LC–NMR) offer significant advantages by enhancing sensitivity, selectivity, and structural elucidation capabilities through integrated separation and detection platforms [81]. By incorporating these techniques into complex natural plant matrices, advanced structural elucidation can be achieved [82].

### 6.5 Methodological Considerations and Analytical Trade‐Offs

Although traditional spectroscopic and chromatographic techniques remain indispensable for flavonoid identification, each methodology has inherent advantages and limitations that influence data interpretation [83]. Spectroscopic approaches, including UV–visible spectroscopy, infrared spectroscopy, NMR, and mass spectrometry (MS), provide rapid insights into the molecular structure and functional group composition. However, these techniques may lack sufficient resolution to reliably distinguish closely related flavonoid isomers with subtle structural differences [76]. In contrast, chromatographic and hyphenated techniques, such as high‐performance liquid chromatography (HPLC), LC–MS, and LC–NMR, offer enhanced sensitivity and superior separation efficiency compared to other techniques. In 2011, Ignat et al., conducted a systematic evaluation of operational trade‐offs in the comparative assessment of analytical methods for polyphenolic characterization. Their study highlighted that colorimetric assay, such as the aluminium chloride method, and HPLC with diode‐array detection (HPLC–DAD) are highly cost‐effective and suitable for high‐throughput subclass profiling. However, these methods often encounter issues such as matrix interference, structural overestimation, and difficulties in resolving co‐eluting isomeric glycosides. In contrast, advanced hyphenated platforms such as liquid chromatography–electrospray ionization–tandem mass spectrometry (LC–ESI–MS/MS) and LC–NMR provide clear structural elucidation and sub‐nanogram detection thresholds necessary for accurate QSAR input descriptors. However, their routine application is limited by high operational costs, significant solvent consumption, complex sample preparation, and the need for specialized expertise [84]. Thus, balancing these methodological parameters is crucial to ensure both analytical rigor and resource efficiency in natural product drug discovery [85]. Recognizing these methodological trade‐offs is essential, as accurate structural characterization forms the foundation for reliable computational modeling, QSAR analysis, and biological evaluation of flavonoids [84]. The key qualitative identification tests, spectroscopic assays, and advanced analytical techniques employed for flavonoid analysis are summarized in Tables 2 and 3.

**TABLE 2:** Chemical tests for flavonoids.

Chemical tests of flavonoids			
Test	Composition	Inference	Reference
Ferric chloride test	5% FeCl_3_	Green precipitate	[86]
Ammonia test	Dilute NH_4_OH + H_2_SO_4_	Yellow color	[71]
Shibata's reaction	HCl + Mg metal	Red/yellow/orange color	[87]
Lead acetate test	10% Pb (CH_3_COO)_2_	Yellow color	[88]
Alkaline reagent test	2% NaOH + HCl	Yellow color	[89, 90]
Pew's test	Zn metal + H_2_SO_4_	Red color	[91]
HCl test	HCl	Red color	[92]
Zinc hydrochloride tests	Zn (metal) + HCl	Magenta color	[93, 94]
Sulphuric acid test	H_2_SO_4_	Orange color	[95]
Shinoda's test	Alcohol + Mg ribbon + HCl	Pink color	[96]

**TABLE 3:** Advantages and disadvantages of various assay techniques.

	Advantage	Disadvantages	
Spectroscopic techniques			
UV–visible spectroscopy	Cost‐effective, quick, and easy to handle	Influenced by temperature and *p*H, non‐selective due to overlapping λ_max_ due to the presence of different flavonoids	[81, 97, 98]
Terahertz	Fast, safe, and non‐destructive	Limited penetration, sensitivity, costly and scattering effects	[83]
IR	Easy, cheap, accurate, no extended sample preparation required, non‐destructive measurement	Need spectrum library and interpretation tool	[99, 100]
NMR	High reproducibility, minimal sample preparation steps, non‐invasive, rapid acquisition of data, high adjustability	High maintenance cost, low sensitivity, limited qualitative results	[101, 102]
Mass	High sensitivity, accuracy	Expensive, isomers can't be distinguished	[103]
Raman	Easy, cheap, not requiring extended sample preparation, suitable for any sample size and type	Need spectrum library and interpretation tool	[99]
Fluorescence spectroscopy	Relevant for some sub‐groups of flavonoids	Additional sample preparation is required to form fluorescence complex	[104]
**Chromatographic techniques**			
TLC	Implementation of multidirectional separation, possibility of long‐term storage of analytical separation	Difficult quantification, affected by environmental conditions, difficult to hyphenate with other techniques	[81, 105, 106]
HPLC	Flexible, reliable characterization and quantification, high separation efficacy, no preliminary derivatization needed	Expensive instrumentation, long detection time	[107]
GC	Precise measurements, no interaction of mobile phase with analyte	Degradation of thermolabile substances, labourious derivatization	[108]
SFC	Short time, no pollution, less wastage of organic solvents, low loss, environmentally friendly, high separation efficacy	High set‐up cost	[107]
CE	High resolution and efficacy, short analysis time required, low sample consumption	Blurring of component zone, sample overloading	[109]

Abbreviations: CE, capillary electrophoresis; GC, gas chromatography; HPLC, high‐performance liquid chromatography; IR, infrared; MS, mass spectrometry; NMR, nuclear magnetic resonance; SFC, supercritical fluid chromatography; TLC, thin‐layer chromatography; UV, ultraviolet.

## 7 Relationship Between Physicochemical Properties of Flavonoids and Their Pharmacokinetics

Like many plant‐derived secondary metabolites, flavonoids occur naturally in both free (aglycone) and glycosylated forms. In general, the biological activity of flavonoids is primarily associated with the aglycone moiety, whereas glycosylation with sugar residues often reduces intrinsic activity and increases molecular polarity. Notably, glycosylated flavonoids may exhibit improved oral absorption compared to their aglycone counterparts; however, absorption efficiency is strongly influenced by multiple physicochemical parameters, including aqueous solubility, p*K*a, molecular size, stereochemical configuration, and lipophilicity [77, 110, 111, 112].

Glycosylation significantly modulates solubility and consequently affects key pharmacokinetic processes, such as absorption, tissue distribution, metabolism, and excretion. These effects highlight glycosylation as a critical structural determinant in optimizing flavonoid pharmacokinetics and should be carefully considered during the rational design and development of flavonoid‐based therapeutic agents [113].

## 8 Mechanism of Action of Flavonoids

The anticancer effects of flavonoids are multifaceted and arise from their ability to concurrently modulate multiple hallmarks of cancer, reflecting a pronounced polypharmacological profile [7]. Rather than acting on a single molecular target, flavonoids exert pleiotropic effects that collectively disrupt tumor cell proliferation, survival, invasion, and resistance to therapy. This multitarget behavior is particularly advantageous in complex malignancies, where signaling pathway redundancy often limits the efficacy of single‐target agents. Flavonoids regulate cell cycle progression by modulating cyclin‐dependent kinases and checkpoint regulators, leading to cell cycle arrest at various phases, including G_0_/G_1_, S, and G_2_/M phases [114]. In parallel, they promote programmed cell death by activating both intrinsic and extrinsic apoptotic pathways, regulating the balance between pro‐ and anti‐apoptotic proteins, inducing caspase activation, and disrupting the mitochondrial membrane potential [115].

Resistance to apoptosis remains a major challenge in effective cancer therapy and is frequently associated with the dysregulation of caspases and B‐cell lymphoma (Bcl)‐2 family proteins. Consequently, strategies aimed at restoring or amplifying pro‐apoptotic signaling represent a critical avenue for anticancer drug development [7, 116]. Notably, flavonoids can overcome apoptotic resistance by inducing autophagic cell death in cancer cells deficient in key apoptotic regulators, such as Bax and Bak [69]. Autophagy‐mediated cytotoxicity triggered by flavonoids is governed by the coordinated regulation of interconnected signaling pathways, including the phosphoinositide 3‐kinase/protein kinase B/mammalian target of rapamycin (PI3K/Akt/mTOR), AMP‐activated protein kinase (AMPK), mitogen‐activated protein kinase (MAPK), Beclin‐1, and Wnt/β‐catenin pathways [8, 117].

**FIGURE 2:**
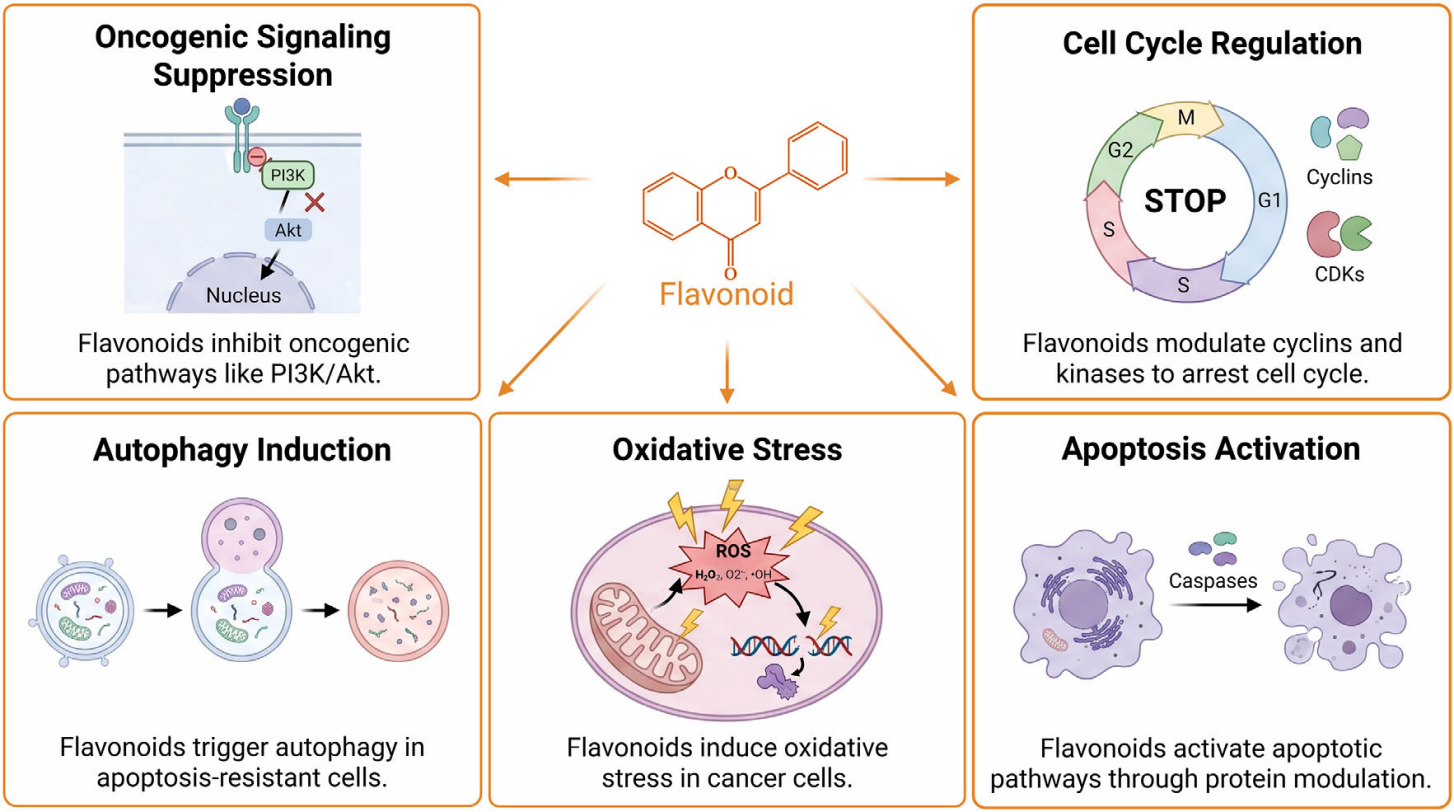
Pleiotropic mechanisms underlying the anticancer effects of flavonoids. Flavonoids modulate multiple cellular processes involved in cancer progression, including suppression of oncogenic signaling pathways such as PI3K/Akt, regulation of cell‐cycle progression, induction of apoptosis, and autophagy, and modulation of oxidative stress, thereby promoting cancer cell death and inhibiting tumor cell proliferation. Created in BioRender. Mishra, A. (2026) https://BioRender.com/gt2gbwz.

Beyond apoptosis and autophagy, flavonoids suppress multiple oncogenic signaling cascades, such as PI3K/Akt, MAPK, nuclear factor kappa B (NF‐κB), and signal transducer and activator of transcription 3 (STAT3), which play pivotal roles in tumor growth, angiogenesis, immune evasion, and metastasis [118]. Among these, the PI3K/Akt pathway is a central regulatory axis in cancer cell survival, metabolic reprogramming, and resistance to therapy (Figure 2). Aberrant activation of the PI3K/Akt signaling pathway promotes uncontrolled proliferation, inhibition of mitochondrial apoptosis, angiogenesis, and metastatic progression [119]. Flavonoids have been shown to attenuate this pathway by inhibiting PI3K activity, reducing Akt phosphorylation, and suppressing downstream mTOR signaling, thereby impairing protein synthesis and tumor cell growth [120]. Inhibition of Akt signaling further facilitates the activation of pro‐apoptotic proteins, enhances mitochondrial cytochrome c release, and triggers the activation of caspase. Additionally, suppression of the PI3K/Akt–mTOR axis contributes to autophagy induction, which may function as a cytotoxic mechanism in apoptosis‐resistant cancer cells. Collectively, these findings position PI3K/Akt signaling as a central node through which flavonoids exert their polypharmacological anticancer effects [121].

**FIGURE 3:**
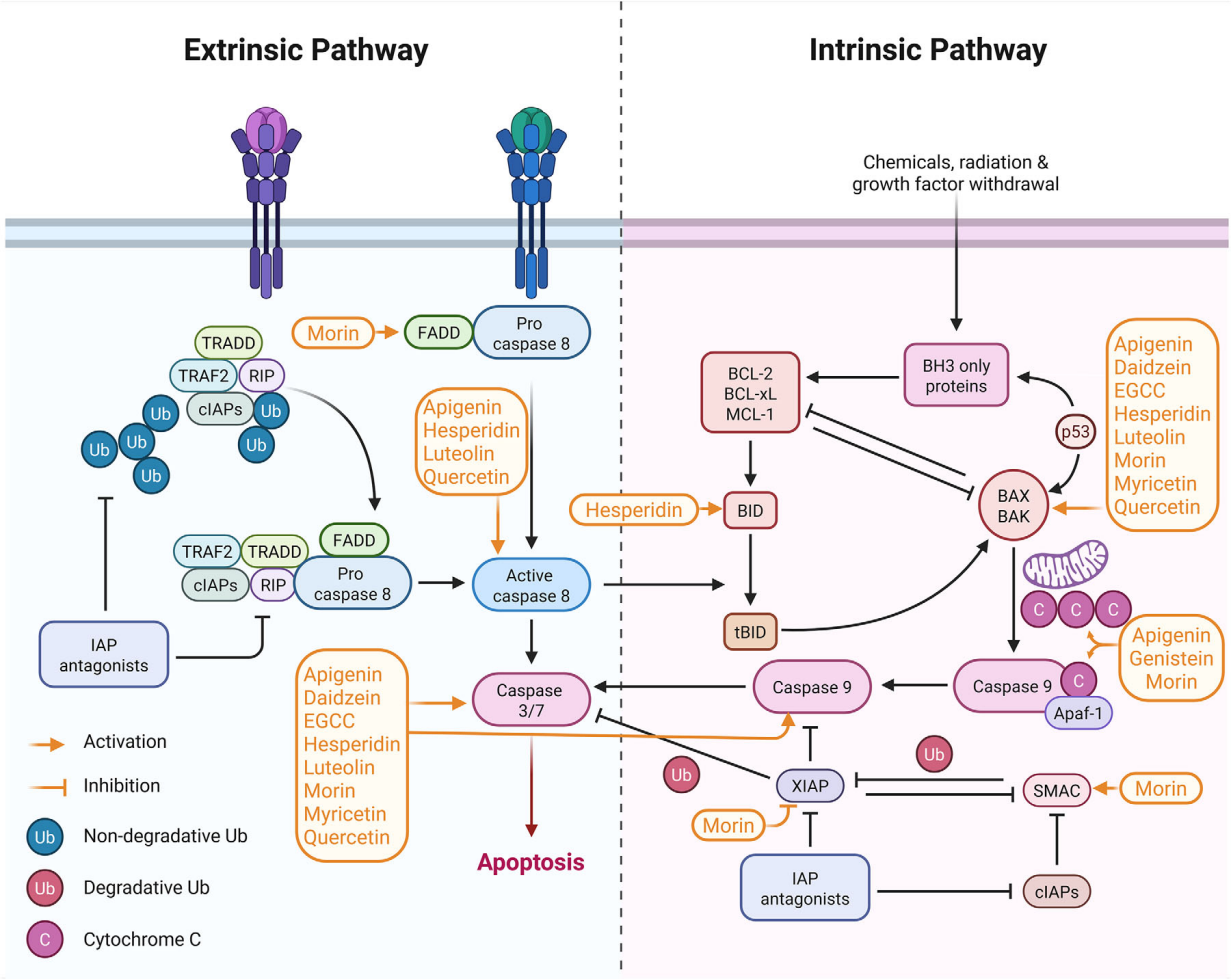
Proapoptotic mechanisms of flavonoids contributing to their anticancer activity. Flavonoids modulate both intrinsic and extrinsic apoptotic pathways by regulating mitochondrial cytochrome c release, apoptotic protease‐activating factor 1 (APAF‐1), B‐cell lymphoma 2 (Bcl‐2) family proteins (Bcl‐2 and Bcl‐xL), inhibitor of apoptosis proteins (IAPs), second mitochondria‐derived activator of caspases (SMAC), truncated Bid (tBid), and tumor necrosis factor (TNF)‐mediated signaling, ultimately promoting caspase activation and apoptosis‐mediated cancer cell death. Created in BioRender. Mishra, A. (2026) https://BioRender.com/a7qx9qn.

Importantly, pathway selectivity and biological responses to flavonoids vary among subclasses and are highly dependent on structural features, underscoring the necessity of integrating mechanistic insights with SAR and QSAR analyses for rational lead identification and optimization. Future mechanistic investigations should emphasize systems‐level approaches, incorporating omics‐based profiling alongside computational modeling to elucidate network‐level effects and support the translation of flavonoid‐based leads into clinically viable anticancer therapeutic programs [122]. Figure 3 provides a schematic overview of the major flavonoid‐mediated anticancer mechanisms.

## 9 Qualitative Structure–Activity Relationship of Flavonoids in Correlation With Anticancer Activity

Quantitative structure–activity relationship (QSAR) modeling has emerged as a powerful computational approach for predicting and rationalizing the anticancer potential of flavonoids by correlating their chemical structures with biological responses [123]. Unlike traditional qualitative SAR analyses, QSAR employs numerical descriptors that quantitatively represent the steric, electronic, hydrophobic, and topological features governing flavonoid–target interactions [33]. These descriptors are derived from two‐dimensional (2D), three‐dimensional (3D), and quantum chemical calculations, enabling a systematic evaluation of how subtle structural modifications influence the antiproliferative activity across diverse cancer models [70]. To provide a systematic framework for evaluating the computational insights, this section is divided into the following subsections.

### 9.1 Physico‐Chemical and Molecular Descriptors Governing Activity

In flavonoid‐based QSAR studies, electronic descriptors such as the highest occupied molecular orbital (HOMO), lowest unoccupied molecular orbital (LUMO) energy gaps, dipole moments, and charge distributions serve as indicators of the capacity of a molecule to engage in π–π stacking, hydrogen bonding, and redox‐mediated interactions with oncogenic targets [124]. Hydrophobic descriptors, including LogP and molecular surface area, are critical determinants of membrane permeability and bioavailability, whereas steric parameters associated with molecular size and shape influence the binding orientation within enzyme active sites or receptor pockets [125]. Collectively, these descriptors explain why variations in hydroxylation, glycosylation, prenylation, and ring substitution patterns result in significant differences in the anticancer efficacy among flavonoid subclasses [126]. Molecular descriptors, which are mathematical representations derived from molecular structures, encode chemical information essential for activity prediction. Currently, over 3000 descriptors, categorized as one‐dimensional (1D), two‐dimensional (2D), and three‐dimensional (3D), are available through dedicated software tools [127]. While 1D descriptors describe basic molecular properties, 2D descriptors capture the topology, size, and electronic features, and 3D descriptors reflect the conformational and spatial characteristics. For instance, semi‐empirical studies on fisetin revealed that its high antioxidant activity is attributable to the low bond dissociation enthalpy of the 3‐OH, 3′‐OH, and 4′‐OH groups, conferring enhanced hydrogen‐donating and radical‐scavenging capacity [128].

### 9.2 QSAR Methodologies, Machine Learning, and Multitarget Integration

Despite their utility in elucidating the structure, QSAR models for flavonoids face notable challenges. The structural redundancy among flavonoids, limited availability of standardized biological datasets, and variability in experimental assay conditions restrict the development of robust and predictive models [129]. Moreover, classical QSAR approaches often assume linear relationships between structure and activity, whereas polyphenolic scaffolds frequently exhibit nonlinear behaviors that cannot be adequately captured by conventional methods. To overcome these limitations, machine learning based QSAR approaches, including random forests, support vector machines, and deep neural networks, are increasingly being employed to address structural complexity and enhance external validation [130]. Future QSAR frameworks for anticancer flavonoids should integrate multitarget datasets, metabolism‐ and toxicity‐related descriptors, and complementary molecular docking and molecular dynamics simulations to evolve QSAR from a correlative tool into a predictive, mechanism‐oriented drug discovery platform [131]. The integration of modern QSAR workflows with machine learning algorithms substantially improves model robustness and facilitates the prediction of multitarget polypharmacology across various cancer cell lines [132].

QSAR modeling, together with molecular docking, constitutes a core component of computer‐aided drug design (CADD) for predicting the biological activity of natural and synthetic compounds [133]. QSAR studies of several flavonoids, including fisetin, were performed using semi‐empirical PM3 and frontal polygon methods, and the model performance was compared using experimental, calculated, and predicted pIC25 values. The superior correlation coefficients obtained with QSAR models highlight the contribution of specific molecular fragments to biological activity [134].

### 9.3 Mechanistic Target Interactivity and Molecular Docking Principles

Molecular docking studies elucidate the mechanistic basis of flavonoid anticancer activity by predicting ligand‐binding modes, interaction networks, and relative affinities toward cancer‐related targets [135]. Docking analysis indicated that fisetin interacts with cyclin‐dependent kinase 6 (CDK6) through hydrogen bonding mediated by hydroxyl groups at the 3′, 4′, and 3 positions, facilitating conformational changes essential for cyclin binding and kinase inhibition [135]. Similarly, QSAR analysis of fisetin against P‐glycoprotein, a key mediator of multidrug resistance, identified solubility, dipole moment, and surface descriptors as critical determinants of inhibitory potency, with predicted IC_50_ values closely matching experimental data [33].

Flavonoids exhibit favorable binding to kinases, transcription factors, and apoptosis‐regulating enzymes owing to their planar aromatic rings and optimally positioned hydroxyl groups, which enable hydrogen bonding and π–π stacking interactions with aromatic amino acid residues [136]. Prenylated flavonoids often display enhanced potency due to their increased hydrophobic surface area, which complements the lipophilic binding pockets of oncoproteins [137]. Nevertheless, docking approaches are constrained by static protein conformations and limitations in scoring functions, which inadequately account for protein flexibility, solvation, and entropic effects [138].

### 9.4 Subclass‐Specific Computational Case Studies and Target Specificity

Extensive *in silico* investigations have been conducted on key flavonoids, including quercetin, kaempferol, hesperidin, apigenin, luteolin, icaritin, and myricetin, to evaluate their interactions with a range of cancer‐associated molecular targets, such as epidermal growth factor receptor (EGFR), mTOR, MAPKs, HDACs, estrogen receptor (ER), and apoptosis‐related proteins. Collectively, these studies underscore the critical role of structural features, particularly hydroxylation patterns, hydrophobic substitutions, and electronic characteristics, in modulating the binding affinity, target selectivity, and predicted biological activity. Selected representative studies on different subclasses are detailed below.

#### 9.4.1 Flavanol

*In silico* analysis of quercetin against several target proteins, including apoptotic regulators, heat shock proteins, cytochrome P450 enzymes, actin, and tyrosine protein kinase Hck, indicated that its anticancer activity is attributed to the presence of a 2‐phenyl‐4H‐benzo[H]chromen‐4‐one pharmacophore [139]. Furthermore, the applicability of quercetin as a lead EGFR inhibitor was revealed through protein‐ligand interaction profiling, which revealed that hydrogen bonding, π–π interactions, salt bridges, and van der Waals forces play critical roles in stabilizing receptor conformations, thus highlighting the promise of quercetin‐based compounds for NSCLC treatment [140].

A 3D‐QSAR model also identified the size and charge requirements for quercetin and its analogs to effectively inhibit NSCLC, providing robust scientific data to move from molecular simulations to clinical trials [141]. Tu et al. conducted *in vitro* structure–activity relationship studies of quercetin and naringenin using the Surflex–Dock program, which provided insights into their binding orientations at DNA sites. Quercetin exhibits strong DNA intercalation ability with the double helix, attributed to the presence of a double bond in ring A, which contributes to structural stability [142]. Rouane et al. established correlations between physicochemical parameters (steric and electronic properties) and the biological activities of quercetin analogs using a multilinear regression (MLR) model. Model validation using the leave‐one‐out (LOO) method, predicted residual sum of squares (PRESS), total sum of squares deviation (SSY), and cross‐validated correlation coefficient (r^2^adj) indicated that increased hydrophobicity negatively influenced the chemotherapeutic activity. A low PRESS/SSY ratio (0.024) confirmed the robustness and reliability of the developed QSAR model [143].

Siniparad et al. evaluated kaempferol as a potential mTORC1 inhibitor using *in silico* molecular docking. Kaempferol exhibited strong binding affinities toward the FRB domain of mTOR (PDB ID: 2FAP; docking score: 27.3) and AKT1 (PDB ID: 3CQU; docking score: 33.4), highlighting its therapeutic potential against hepatocellular carcinoma through modulation of the mTOR signaling axis [144]. Further docking analysis using AutoDock (v4.2.6) revealed stable interactions between kaempferol with human ERα and HER4/ErbB4 kinase, yielding favorable binding energies of −7.39 and −8.18 kcal/mol, respectively. These findings underscore the potential of kaempferol as a viable lead scaffold for multi‐target breast cancer therapeutics [145]. In addition, molecular docking and simulation studies targeting xanthine oxidase (XO) have demonstrated that kaempferol binds efficiently within the hydrophobic catalytic pocket containing the molybdenum center. The most stable binding pose exhibited an estimated binding energy of −4.71 kcal/mol and was stabilized by hydrogen bonding and hydrophobic interactions involving critical residues, such as Phe1009, Phe914, Asn768, and Leu1. These interactions suggest that kaempferol may inhibit XO activity by occupying the catalytic site and inducing conformational constraints, thereby modulating purine metabolism and oxidative stress–related pathways [146].

Density functional theory (DFT) calculations were employed to elucidate the structure–activity relationship of myricetin, an antioxidant molecule. Calculations performed at the M06‐2X/6‐31++G (d, p) level highlighted the pivotal role of the 4′‐hydroxyl group in stabilizing ligand–target interactions *via* weak hydrogen bonding, which was preserved upon complex formation [147].

Further molecular docking and *in silico* pharmacokinetic studies demonstrated that myricetin and its analogs exhibit a strong binding affinity for phosphoinositide‐dependent kinase‐1 (PDK‐1), a central regulator of the PI3K/AKT signaling pathway that is frequently dysregulated in cancer. The tight accommodation of ligands within the catalytic site supports their potential as PDK‐1 inhibitors [148]. Additionally, docking studies using AutoDock 4.2 examined the interaction of myricetin with thiol isomerase enzymes, including endoplasmic reticulum protein‐5 (ERp5) and protein disulfide isomerase (PDI). Fluorescence quenching analyses indicated ligand interactions with tryptophan residues, corroborating the predicted binding modes and reinforcing the relevance of myricetin as a bioactive compound with anticancer potential [149].

#### 9.4.2 Flavone

Comprehensive *in silico* analyses were conducted to assess the anticancer potential of apigenin and related flavonoids against multiple molecular targets implicated in tumor progression, including cyclin‐dependent kinase‐2 (CDK‐2), topoisomerase IIα, and vascular endothelial growth factor receptor‐2 (VEGFR‐2). Integrated computational approaches encompassing ADME profiling, PASS prediction, pharmacophore modeling, DFT calculations, molecular docking, and molecular dynamics (MD) simulations have demonstrated that these phytoconstituents exhibit comparable inhibitory profiles across the selected targets, supporting their multitarget therapeutic relevance [150].

The interactions of apigenin and luteolin with class I histone deacetylases (HDACs) was further evaluated using extra‐precision docking, MM‐GBSA free energy calculations, and MD simulations. Both compounds showed stable binding to the catalytic pockets of HDAC2 and HDAC8. While apigenin displayed consistent affinity across isoforms, luteolin exhibited more favorable binding free energy values, likely reflecting subtle structural differences that influence isoform selectivity. These findings support the potential of flavonoids as natural scaffolds for HDAC‐targeted anticancer strategies [151].

In a simulation‐based docking study targeting the EGFR L858R mutant associated with non‐small cell lung cancer (NSCLC), 45 phytochemicals, including luteolin glycoside, were screened. Luteolin and epicatechin gallate formed stable interactions with the key residue, Met793. Subsequent MD simulations confirmed the stability of these complexes, as evidenced by sustained hydrogen bonding, favorable radius of gyration, and minimal conformational deviations, indicating their potential relevance in EGFR‐driven NSCLC [152].

Extra‐precision docking studies of luteolin and its analogs against tankyrase‐II revealed that luteolin formed multiple hydrogen bonds within the active site, resulting in enhanced binding affinity. Favorable pharmacokinetic predictions and SiteMap analyses further supported optimal hydrogen bonding and hydrophobic interactions, highlighting luteolin as a promising lead compound for further investigation [153].

The antiglioma potential of luteolin was explored using a combination of network pharmacology and molecular docking approaches. Target mapping using the KEGG and Gene Ontology databases identified AKT1, JUN, MAPK1, MAPK3, ALB, and TNF as key nodes. Docking and MD simulations conducted using the AMBER14 platform demonstrated stable interactions between luteolin and these targets, suggesting its ability to modulate multiple signaling pathways involved in glioma pathophysiology [154].

#### 9.4.3 Flavanone and Isoflavone/Prenylated Derivatives

Selim et al. performed *in silico* docking studies of bioflavonoids, including hesperidin, to evaluate their interactions with MAPKs. Using the FlexX module of the LeadIT software, chrysophanol, physcion, hesperidin, and curcumin demonstrated stable binding within the p38α MAPK active site without steric clashes, suggesting their potential efficacy against hepatitis‐associated hepatocellular carcinoma [155]. Additionally, molecular docking of hesperidin against key inflammatory mediators—tumor necrosis factor (TNF)‐α, interleukin (IL)‐1β, IL‐6, and NOS using AutoDock revealed strong binding affinities mediated by hydrogen bonding, π–π, π‐alkyl, and van der Waals interactions with critical amino acid residues. These multitarget interactions support the anti‐inflammatory and anticancer potential of hesperidin through the modulation of MAPK and cytokine‐driven signaling pathways [156].

The ER modulatory activity of icaritin and its derivatives was investigated using molecular docking studies. Wushanicaritin exhibited favorable binding affinities toward ER‐α and ER‐β, whereas icaritin showed a particularly strong affinity for ER‐β. In contrast, hydrous icaritin demonstrated weaker interactions. These findings suggest that icaritin derivatives may exert anticancer effects through selective modulation of ER [157].

Experimental validation using a biotin‐based affinity assay demonstrated that icaritin interacts with IκB kinase‐α (IKK‐α) *via* critical cysteine residues, resulting in the suppression of NF‐κB signaling and downstream p65 activation. This inhibition was associated with the reduced expression of immune checkpoint–related proteins, supporting the anti‐inflammatory and anticancer potential of icaritin [158].

#### 9.4.4 Experimental Corroboration and Validation Strategies

To facilitate clinical translation and avoid computational artefacts, it is essential that *in silico* predictions are validated using direct biochemical, cell‐based, or biophysical assays [159]. A study was conducted to experimentally validate the interaction between icaritin and IκB kinase‐α (IKK‐α) through critical cysteine residues *via* biotin‐based affinity assay. This interaction suppresses NF‐κB signaling and subsequent p65 activation. This inhibition is correlated with a decreased expression of immune checkpoint‐related proteins, thereby supporting the anti‐inflammatory and anticancer potential of icaritin [158].

Overall, the integration of QSAR modeling with molecular docking, molecular dynamics simulations, and quantum chemical calculations provides a comprehensive framework for understanding the flavonoid structure–activity relationships. Such integrative *in silico* strategies hold significant promise for the rational optimization of flavonoid scaffolds and their translation into effective anticancer therapeutics.

## 10 Methodological Limitations of QSAR Models and Their Translational Challenges

This section systematically categorizes the reporting practices used to critically evaluate the reliability and translational value of QSAR models developed for the prediction of anticancer activity of flavonoids. The categorization was based on statistical metrics, depth of validation (distinguishing between *in silico* and *in vitro*/*in vivo* methods), nature of application (prospective versus retrospective), and instances of computational–experimental inconsistency.

### 10.1 Statistical Robustness, Internal Metrics, and Subclass‐Specific Limitations

Statistical parameters such as the coefficient of determination (R^2^), cross‐validated coefficient (Q^2^), root mean square error (RMSE), and external test‐set validation are routinely employed to assess the robustness and predictive performance of QSAR models developed for flavonoid‐based anticancer agents [12, 122]. In many reported studies, acceptable R^2^ and Q^2^ values indicate reasonable internal consistency; however, meaningful cross‐comparison among models remains challenging owing to heterogeneity in descriptor selection, dataset size, endpoint definition, and biological assay conditions [160]. Notably, QSAR models constructed using structurally constrained flavonoid subsets often demonstrate high statistical accuracy but suffer from limited chemical space coverage, thereby restricting their generalizability when applied to structurally diverse flavonoid libraries [161].

Comparative QSAR analyses across flavonoid subclasses suggest that flavonols and prenylated flavonoids exhibit stronger correlations with anticancer activity than glycosylated derivatives. This trend is primarily attributed to differences in lipophilicity, electronic delocalization, and steric factors that govern target engagement and cellular permeability [162]. Despite these insights, QSAR investigations in this domain remain largely descriptive and case‐specific, with limited emphasis on extracting transferable structure–activity relationship. Consequently, there is a growing need for cross‐study and meta‐analytical approaches that identify consistent descriptor–activity trends across flavonoid classes, rather than relying on isolated correlations [163].

### 10.2 Differentiation of *In Silico* Prediction Versus Multilevel Experimental Validation

A critical distinction must be drawn between studies that rely exclusively on theoretical docking scores or 2D/3D‐QSAR predictions and those that are substantiated by *in vitro*, cellular, biochemical, or *in vivo* xenograft studies.

Several intrinsic limitations constrain the translational relevance of QSAR models. Overfitting, reliance on small or biased datasets, and inadequate external validation remain persistent pitfalls that can inflate the apparent predictive power [160]. Moreover, QSAR predictions are frequently not supported by complementary experimental validation, limiting their applicability beyond hypothesis generation. Even statistically robust *in silico* models often fail to translate into clinically viable anticancer candidates because of factors such as poor oral bioavailability, rapid metabolic degradation, and off‐target toxicity, which are parameters that are insufficiently captured by conventional QSAR descriptors [163]. Addressing these challenges will require integrative modeling strategies that combine QSAR with experimental ADMET profiling, mechanistic cellular assays, and iterative model refinement to enhance predictive accuracy and translational potential [164].

While QSAR and molecular docking offer valuable insights into the structure–activity relationships of flavonoids, their scientific utility ultimately depends on experimental confirmation [165]. Encouragingly, several studies have demonstrated concordance between QSAR‐derived descriptors, such as lipophilicity, electronic distribution, and hydrogen‐bonding capacity, and experimentally observed antiproliferative or pathway‐specific effects, supporting the role of computational approaches in early‐stage lead prioritization [166].

### 10.3 Retrospective versus Prospective Application and Computational Inconsistencies

A primary limitation of contemporary computational workflows is their predominant dependence on retrospective validation, which involves testing models against established, pre‐existing compound sets. This approach contrasts with genuine prospective virtual screening, which aims to identify novel active hits before synthesis or extraction and subsequent experimental testing. Nevertheless, many QSAR‐identified hits are evaluated retrospectively rather than prospectively, limiting their impact on rational drug development. Discrepancies between *in silico* predictions and experimental outcomes often arise from factors inadequately represented in computational models, including limited cellular uptake, metabolic variability, and context‐dependent signaling pathways [167].

### 10.4 Translational Implications

Taken together, these considerations underscore the importance of interpreting QSAR outputs as hypothesis‐generating rather than as definitive predictors of anticancer efficacy. Strengthening iterative workflows that integrate QSAR modeling with experimental validation, ADMET assessment, and mechanistic studies will be essential for improving translational reliability and advancing flavonoid‐based anticancer candidates toward clinical applicability [168].

## 11 Conclusion

This review provides a comprehensive synthesis of recent advances in the identification, characterization, computational modeling, and mechanistic evaluation of flavonoids as anticancer agents. By integrating classical phytochemical approaches with advanced spectroscopic, chromatographic, and hyphenated analytical techniques, the structural diversity of flavonoids has been systematically elucidated and correlated with their biological activity. Importantly, QSAR and molecular docking analyses highlighted that structure‐dependent electronic, steric, and lipophilicity parameters critically govern target engagement, pathway modulation, and anticancer efficacy. Mechanistic evidence further demonstrates that flavonoids exert their anticancer effects through the coordinated regulation of apoptosis, autophagy, oxidative stress, and key oncogenic signaling cascades, including PI3K/Akt, MAPK, NF‐κB, and STAT3. Collectively, these findings underscore the therapeutic potential of flavonoids as multitarget anticancer agents and emphasize that their rational development is best achieved through an integrated framework that combines computational predictions with experimental validation.

## 12 Future Prospects

Future research should increasingly focus on tightly integrated QSAR–experimental pipelines to accelerate the translational development of flavonoid‐based anticancer leads. Rather than relying on single‐endpoint activity models, next‐generation QSAR approaches should adopt multi‐endpoint, pathway‐centric frameworks capable of capturing the inherent polypharmacology of flavonoids and their coordinated regulation of the PI3K/Akt, MAPK, NF‐κB, and STAT3 signaling networks. The integration of QSAR with molecular docking, molecular dynamics simulations, and experimentally validated ADMET profiling is essential for improving predictive reliability and minimizing late‐stage attrition.

In parallel, machine learning driven QSAR models trained on curated, chemically and mechanistically annotated datasets hold significant promise for efficiently exploring the flavonoid chemical space and identifying optimized analogs. Coupling these computational insights with high‐content phenotypic screening, omics‐based validation, and network‐level analyses will bridge the gap between *in silico* predictions and their biological relevance. Such interdisciplinary, data‐driven strategies are poised to substantially advance the preclinical optimization and translational progression of flavonoid‐derived anticancer agents.

## Author Contributions

**Mukta Gupta**: investigation, methodology, resources, validation, formal analysis, Writing – original draft. **Shanu Priya**: investigation, writing – original draft, validation, methodology, formal analysis, resources. **Javed Ahmad**: investigation, methodology, resources, writing – review and editing, validation, formal analysis. **Kasim Sakran Abass**: investigation, methodology, validation, resources, formal analysis, writing – original draft. **Awanish Mishra**: conceptualization, investigation, methodology, resources, validation, formal analysis, software, visualization, supervision, project administration, data curation, writing – original draft, writing – review and editing.

## Conflicts of Interest

The authors declare no conflicts of interest.

## Funding

The author has nothing to report.

## Data Availability

Data sharing not applicable to this article as no datasets were generated or analyzed during the current study.
